# Clinical Characteristics and Outcome Related to Blood Eosinophilic Chronic Obstructive Pulmonary Disease (COPD) Patients

**DOI:** 10.7759/cureus.27998

**Published:** 2022-08-14

**Authors:** Amr Albanna, Fatimah M Almuyidi, Noura F Beitar, Amal S Alshumrani, Ziyad F Al Nufaiei, Rouzana Khayat, Majid Althaqafy, Hawazen I Abdulmannan

**Affiliations:** 1 Pulmonology, King Abdulaziz Medical City-Western Region, Jeddah, SAU; 2 Respiratory Therapy, King Saud Bin Abdulaziz University for Health Sciences, Jeddah, SAU; 3 Respiratory Therapy, King Abdullah International Medical Research Center, Jeddah, SAU; 4 Research Unit, King Saud Bin Abdulaziz University for Health Sciences, Jeddah, SAU; 5 Research Unit, King Abdullah International Medical Research Center, Jeddah, SAU; 6 Biostatistics, King Saud Bin Abdulaziz University for Health Sciences, Jeddah, SAU; 7 Biostatistics, King Abdullah International Medical Research Center, Jeddah, SAU

**Keywords:** long- β-agonist, inhaled corticosteroids, emergency room, exacerbation, chronic obstructive pulmonary disease

## Abstract

Introduction

The term chronic obstructive pulmonary disease (COPD) refers to a range of illnesses that impair breathing and airflow. Clinical history of COPD is further impacted by frequent exacerbations known as acute exacerbation COPD in which these specific symptoms worsen contributing to emergency room (ER) visits and hospitalization. Blood eosinophils are a crucial indicator of therapy effectiveness and exacerbation rate. The role of blood eosinophils as a biomarker for treatment, response, exacerbation risk, inflammation, and other symptoms in COPD patients is implemented by the Global Initiative Obstructive Lung Disease (GOLD) as guidelines.

Objective

To determine the clinical characteristics and outcomes related specifically to eosinophilic COPD Patients.

Methodology

This is a retrospective single-center study of all AECOPD presented at ET between 2018 to 2019. A total of 120 patients were included. Patients were divided into two groups depending on blood eosinophil count: high (>300cells/µL) and low (<300cell/µL). Finally, Binary logistics regression was performed to determine correlations between clinical characteristics and eosinophil count levels.

Results

In the high eosinophil patients’ group: none of the independent variables were statistically significant. However, in the low eosinophil patients’ group: ER visits, lung disease, and symptomatic exacerbation made a statically significant contribution to the model (*p*-value of .008, .01, .001) respectively.

Conclusion and recommendation

The higher eosinophil levels showed better clinical outcomes compared to lower eosinophil levels. Increasing the level of symptomatic AECOPD episodes in low eosinophil levels may be linked to the onset of bacterial infection and airway inflammation. The study further recommends a prospective cross-sectional multi-center study which includes a follow-up of the patients to assess the number of exacerbations after initial treatment

## Introduction

Chronic obstructive pulmonary disease (COPD) is a major community health issue globally. Currently, COPD is the fourth leading cause of death in the world and is projected to hit the third level within 10 years as the attempts to avoid, recognize, diagnose and manage patients internationally are inadequate [1]. COPD is a widespread, preventable, and treatable disease identified by chronic respiratory symptoms and impairment of airflow due to airway and/or alveolar disorders, typically triggered by excessive exposure to noxious particles or gases. COPD's clinical history is also influenced by frequent exacerbations in which these particular signs escalate dramatically, contributing to emergency department visits and hospitalization [2]. The calculation of blood eosinophils is now an essential biomarker for exacerbation rate and management response [3]. Blood eosinophils' potential function is focused primarily on data obtained from retrospective examinations that showed a correlation between increased blood eosinophils and the risk of exacerbation. Such findings are not verified by other publications, yet. However, much remains to be known about the association between an elevated number of blood eosinophilia and their conflicting impact on the status of COPD patients. Interest in the application of blood eosinophils as a biomarker in predicting the treatment response, exacerbations risk, inflammation reversibility, and other clinical symptoms in COPD patients tends to grow, with guidelines on their adjunctive function implemented in the Global Initiative for Chronic Obstructive Lung Disease (GOLD) management algorithm [4]. As the diagnostic criteria for beginning therapy with inhaled corticosteroids (ICS)/long- β-agonist (LABA), the 2019 (GOLD) care recommendations suggested blood eosinophil counts of 300 cells·μL−1 in healthy COPD [5]. In 2014, a study published by European Respiratory Journal showed that 37% of COPD patients with elevated rates of eosinophilic blood correlated with enhanced intake of corticosteroids [6]. Measurements of the blood eosinophil can be effective in targeting patients for different therapeutic strategies. Another recent systematic review and meta-analysis that had been done in London confirmed the ICS' independent role in rising the probability of COPD exacerbation over a number of eosinophil levels in the blood [7]. Talking about Eosinophil level and exacerbation rate, a study was established in 2017 including 8318 subjects noticed that elevated blood eosinophil levels (almost 0.45109/L) were coupled with a higher risk of worsening in patients with controlled COPD the year after [8]. They concluded that the enhanced level of exacerbation with an elevated eosinophil count was severely restricted to ex-smokers and ICS patients. According to previous studies, approximately 60% of COPD patients who have a blood eosinophil count of ⩾2% are more susceptible to high exacerbation risk [9-11]. However, few studies have denied the utilization of peripheral eosinophil calculation as a threshold in COPD management. A study done in 2017 with a total of 7,245 patients were overwhelmingly male (57.1%), white (71.8%), frequently ongoing or former smokers (75.4%), 19.0% had eosinophilic counts greater than or equivalent to 300 cells/mm3, has confirmed that peripheral eosinophil is an independent factor for long term adverse events [12]. Another observational, retrospective, population-based longitudinal follow-up research done in Spain concluded that the findings did not endorse the usage of eosinophils as a valid biomarker of the likelihood of COPD exacerbation by dividing 80% of the population into four subgroups [13]. Also, some trials have confirmed that peripheral eosinophils level seems like a reliable biomarker for estimating the reversibility of inflammation and the sensitivity of COPD patients to ICS [14-16]. Although this method has been endorsed for many prospective trials of predetermined subgroup analyzes, concerns remain as to whether this latest appraisal technique can be integrated into COPD's real-life management, and further work is required to confirm its incorporation into clinical practice. There is a shortage of evidence to justify the usage of biological therapy in eosinophilic COPD phenotypes. In this study, we aimed to identify whether the exacerbation rate and other clinical characteristics are correlated with high eosinophil count versus low eosinophil count in COPD patients.

## Materials and methods

Study design

A retrospective descriptive research design was chosen to analyze the patients’ records to extract all COPD cases presented to the emergency oom (ER). Prior to the initiation of the study, approval was obtained from the Institutional Review Board.

Target population

A consecutive sampling technique was used which involved all eligible patients during the study period who met the inclusion criteria. Three hundred patients were identified over the period of 2018 through 2019. Only 120 patients were included in this study due to the inclusion criteria: 40 years and above COPD primary cause of the ER visit is the exacerbation only. In addition, those patients who were diagnosed with other chronic respiratory diseases were excluded.

Data collection

COPD was defined according to the Global Initiative for Obstructive lung diseases as an irreversible airflow limitation following bronchodilator therapy. Patient charts were reviewed electronically by utilizing the BESTCare system. The patient's charts were reviewed for admission rate, ER visit, exacerbation rate, smoking history, and other demographic characteristics such as gender, and clinical presentation. The researchers classified the patients into two groups based on eosinophil count: high eosinophil count (>300 cells/µL) and low eosinophil count (<300 cells/µL). The outcome variables included age, gender, smoking history, lung comorbidities, and other chronic diseases such as hypertension (HTN) and diabetes mellitus (DM), or treated with inhaled corticosteroids (ICS), systemic corticosteroids (SCS), long-acting muscarinic antagonist (LAMA), or long-acting beta-agonist (LABA). Then, the data obtained was loaded into a Microsoft Excel sheet and secured affordable only to investigators.

Data analysis

SPSS statistical software version 27 was used to analyses data. The proportion and mean for dichotomous categories and continuous variables, respectively, were measured to describe subjects’ characteristics. In addition, Binary logistics regression is used to find correlations between clinical characteristics and eosinophil count levels. Statistical significance was determined using the 95% confidence interval and p-value of 0.05.

## Results

Demographic profile

The initial data extraction included 300 patients between 2018 and 2019. Only 120 patients met the inclusion criteria, so the others were excluded immediately. Of the total number of patients, 76.7% were male. The mean age of all patients was 70.6%. 45.8% were non-smokers. 34.2% of the patients quit smoking while 20% of the patients were presently smoking. Most of the COPD patients (57.5%) were suffering from both hypertension (HTN), and diabetes (DM). At the same time, only 20.8% of the COPD patients were suffering from HTN only. A few patients (16.7%) had no history of any chronic illness accompanied by COPD. Out of 120 COPD patients, 70% were symptomatic, 50% were treated with ICS, 41.7% were treated with SCS, 44.2% were treated with LAMA, and 6.7% were treated with LABA. The mean of low eosinophil count was 49.7/mm3 and for high eosinophil count was 549.7/mm3 (Table 1).

**Table 1:** Clinical Characteristics of Patients

Characteristics	Eosinophil < 300 N = 80	Eosinophil ≥ 300 N= 40
Female sex (%)	19.8	34.5
Smoking history (%)	52.8	58.6
Current smoker (%)	18.7	24.1
Pulmonary comorbidity (%)	48.4	72.4
Diabetes (%)	5.5	3.4
Hypertension (%)	17.6	31
Symptomatic (%)	69.2	72.4
Exacerbation rate	65.9	72.4

Low eosinophils count (<300 cells/µL)

Binary logistics regression was performed. The full model containing all predictors was statistically significant, χ² (13, N=80) = 67.162, p<0.001, indicating that the model was able to distinguish between patients who reported exacerbation and who did not, with respect to the clinical characteristics. The model as a whole explained between 56.8% (Cox and Snell R Square) and 78.7% (Nagelkerke R square) of the variance in exacerbation, and correctly classified 91.3% of cases. Hosmer and Lemeshow Test show a p-value of 0.993 which represent that the model is very efficient in the analysis as being greater than 0.05.

Only three of the independent variables - ER visit, lung disease, and symptomatic made a unique statistically significant contribution to the model. The strongest factor for exacerbation was symptomatic with an odd ratio of 432.008. This indicates that patients who were symptomatic were 432 times more likely to have higher exacerbation than those who weren’t symptomatic. The odds ratio for an ER visit is 0.488, meaning that patients were 0.488 times less likely to have exacerbation for every additional visit. Finally, the odds ratio for lung disease is 91.790, which indicates that patients with comorbidity were 91 times more likely to have exacerbation than those who have only COPD. 

High eosinophils count (>300 cells/µL)

Binary logistics regression was performed. The full model containing all predictors was statistically significant, X2(13, N=40) = 48.869, p< 0.001, indicating that the model was able to distinguish between patients who reported exacerbation and who did not, with respect to the clinical characteristics. The model as a whole explained between 70.5% (Cox and Snell R Square) and 100% (Nagelkerke R square) of the variance in exacerbation, and correctly classified 100% of cases. Hosmer and Lemeshow Test show a p-value of 1.000 which says that the model is very efficient in the analysis as being greater than 0.05. However, none of the independent variables made a unique statistically significant contribution to the model.

## Discussion

A clinically effective biomarker, whether for acute exacerbations of COPD (AECOPD) or other disorders, should ideally represent disease activity consistently and precisely. The test should be functional and consistent in a range of COPD patients and cohorts with the least amount of invasiveness to the patient [1]. Therefore, the aim of the study was to evaluate the utility of blood eosinophil count in COPD patients with regard to its correlation to the exacerbation rate and other clinical characteristics. The present study revealed that the prevalence of DM in study cohorts was 57.5% which is considerably higher than the average world prevalence of DM of 9.3% [2]. COPD is known to be a novel risk factor for the development of DM due to multiple pathophysiological changes [3]. It is also described that nearly half of all COPD patients have other medical conditions that are frequently associated with DM such as HTN and higher cholesterol which is evident in the findings of this study [4,5]. In this study, we found that patients with higher eosinophil levels experienced better clinical outcomes. According to our retrospective analyses, patients with non-eosinophilic exacerbations (<300 cells/µL), were statistically significant for patients who had lung disease. There is limited research on the relationship between blood eosinophil counts and structural alterations in the COPD lung. Lung disease and blood eosinophil count in COPD may be related as a prior study by Wu et al., found patients with rapid lung function decrease had more severe symptoms and lower levels of circulating blood eosinophils [6]. In this study patients categorized in the low blood eosinophil group exhibited a higher rate of symptomatic AECOPD episodes with decreased probability of ER visits prior to initial exacerbation. AECOPD has been associated with systemic inflammation which has been linked to bacterial infection and airway inflammation. A study conducted by Chang et al. reported that 31.1% of patients were potentially positive microorganisms (PPM)-positive at the admission of which 50% remained PPM-positive categorizing them as a bacterial persistence group. The study concluded bacterial infections are positively associated with exacerbation frequencies highlighting the role of bacteria in causing inflammation and exacerbation [7]. Additionally, a longitudinal observational study found that blood eosinophil counts below 100cell/µL were linked to an increased risk of pneumonia and persistent bacterial airway infection. These results show that COPD patients with low eosinophil levels are more susceptible to bacterial airway infection, although the processes underlying these results have not been defined [8]. Enhanced eosinophilic lung inflammation and other biological characteristics are linked to higher blood eosinophil counts. This study showed a greater number of ex-smoker patients were present in the high eosinophilic count (>300/µL) compared to the low eosinophilic count (<300/µL). Similarly, a larger number of current smoker patients were in the higher eosinophilic count group compared to the low eosinophilic count group. However, was no difference in risk estimated when smoking status was analyzed for significance. In a posthoc analysis of three studies, Bafadhel et al. reported in Lancet Respiratory Medicine that smoking status was an independent predictor of responsiveness to budesonide (ISC) in COPD patients and that there was a significant interaction between eosinophil count, therapy, and smoking status [9]. The exacerbation rate was found to be independent of eosinophil count in former smokers, but increased eosinophil count was associated with an increased risk of exacerbation in current smokers. On the other hand, a study of 2400 current or former smokers found no link between peripheral blood eosinophilia (>300 cells/µL) and exacerbation risk, though there was a link between sputum eosinophilia and overall COPD-related outcomes [10]. Similarly, a study in France analyzed 458 patients with COPD and a history of current or previous smoking, and no relationship between exacerbation rate and increasing eosinophil counts above the 2% threshold could be demonstrated [11]. The previously reviewed evidence served as the foundation for the GOLD 2020 recommendations to use blood eosinophil counts as a biomarker to guide ICS treatment in COPD patients with a history of exacerbations [12,13]. As part of a precision medicine approach, these recommendations combine clinical information and patient history collection information on exacerbation risk with biological data such as complete blood count particularly blood eosinophils to maximize the potential benefits [14-16]. 

Strengths and limitations 

The strengths of the study include the collected data from patients presented to the ER displaying a view of the current demographic and clinical characteristics of the COPD patients and the subsequent ER management and implementations of the therapies. However, there were some limitations of the study which included difficulties in ruling out pre-hospitalization medication use among the patients. Pre-hospitalization corticosteroid administration can affect the blood eosinophil count which may be the result of the relatively low prevalence of the high eosinophilic count group of patients. In addition, due to the nature of respective studies, some confounding variables such as pulmonary function test, medication treatment after discharge, and eosinophil count after treatment of AECOPD were also not available and subsequently not analyzed in the study which may present as a bias of results to some extent. Another study limitation is that the results obtained were from a single-center study of patients presented at the ER for AECOPD. The results may not be generalizable to the entire nation's COPD population. Further multicenter studies are very necessary to further elaborate these study results. 

## Conclusions

Blood eosinophil counts are being utilized more frequently in clinical practice to assist in making clinical decisions about the use of ICS. For the best selection of combination inhalers to be used for exacerbation prevention, GOLD recommendations put a strong emphasis on integrating clinical data with blood eosinophil counts. The increased effectiveness of ICS at high blood eosinophil count may have a pathophysiological explanation that eosinophilic AECOPD patients have inflammation. Therefore, reduced blood eosinophil counts are associated with a high risk of chronic bacterial infection. The management of AECOPD lies in the complex relationship between blood and sputum eosinophil count, ICS response, and persistent pathogens in the respiratory tract. The study further recommends a prospective cross-sectional multi-center study that includes a follow-up of the patients to assess the number of exacerbations after initial treatment. This study would be able to report the long-term effects of ISC usage among the eosinophilic and non-eosinophilic AECOPD.
